# Aguamiel, a Traditional Mexican Beverage: A Review of Its Nutritional Composition, Health Effects and Conservation

**DOI:** 10.3390/foods14010134

**Published:** 2025-01-06

**Authors:** Alma Delia Noriega-Juárez, Libier Meza-Espinoza, María de Lourdes García-Magaña, Rosa Isela Ortiz-Basurto, Martina Alejandra Chacón-López, Luis Miguel Anaya-Esparza, Efigenia Montalvo-González

**Affiliations:** 1 Laboratorio Integral de Investigación en Alimentos, Tecnológico Nacional de México Instituto Tecnológico de Tepic, Tepic 63175, Nayarit, Mexico; aldenoriegaju@ittepic.edu.mx (A.D.N.-J.); mgarciam@tepic.tecnm.mx (M.d.L.G.-M.); riobasurt@ittepic.edu.mx (R.I.O.-B.); mchacon@ittepic.edu.mx (M.A.C.-L.); 2 Dirección de Ciencias Agropecuarias, Universidad Tecnológica de la Costa, Santiago Ixcuintla 63300, Nayarit, Mexico; 3 Centro de Estudios para la Agricultura, la Alimentación y la Crisis Climática, Centro Universitario de los Altos, Universidad de Guadalajara, Tepatitlán de Morelos 47620, Jalisco, Mexico; luis.aesparza@academicos.udg.mx

**Keywords:** aguamiel, nutrients, prebiotic, microorganisms, shelf life

## Abstract

Aguamiel is the sap extracted from various species of maguey (*Agave* spp.). This liquid is highly prized in central Mexico for its pleasing sensory qualities and nutritional value. Understanding the composition of aguamiel is crucial as it may offer beneficial effects for human health. Reports have indicated its significance as a source of essential amino acids, vitamins, minerals, and fructooligosaccharides with prebiotic potential. Additionally, aguamiel can harbor diverse microorganisms, including lactic acid bacteria (*Lactococcus* and *Leuconostoc* spp.) and yeasts, contributing antioxidant, nutritional, prebiotic, and probiotic properties. However, aguamiel is prone to rapid fermentation due to its nature, which can alter its sensory and nutritional characteristics. This review provides insight into the broad nutritional composition, microbial diversity, and metabolites beneficial to the human health of fresh aguamiel. At the same time, it reviews the technologies applied to aguamiel to preserve its nutritional properties and functional metabolites and extend its shelf life. Thus, the data included in this document may lead to greater beverage consumption and further research to find new conservation alternatives that change its organoleptic and functional properties as little as possible.

## 1. Introduction

In Mexico, aguamiel is considered a traditional beverage, consumed mainly in the states of Hidalgo, Tlaxcala, and Puebla; there is evidence that it has been part of the diet of the inhabitants of these regions since ancient times [1]. This unique beverage is derived from the sap of maguey (*Agave* spp.), a plant with long-held cultural and economic significance in Mexico [2]. The production of aguamiel involves tapping the maguey plant to collect its sap [3]. This process is typically carried out by skilled artisans who have learned traditional techniques from generations of their ancestors [4]. Aguamiel is a slightly sweet and effervescent liquid commonly consumed fresh, and it offers a unique combination of flavors and nutritional properties that have aroused the interest of researchers and consumers alike. It is also a key ingredient in pulque, a fermented beverage with a slightly alcoholic taste [5]. In addition to its culinary applications, aguamiel has been traditionally used for medicinal purposes and as a source of nourishment [6].

In recent years, aguamiel has gained attention for its potential health benefits and role in promoting cultural heritage [7]. Therefore, it is essential to explore the nutritional composition of aguamiel, highlighting its primary nutrients and bioactive compounds. In addition, the potential health effects associated with aguamiel consumption are discussed in this review, including its antioxidant properties, antimicrobial activity, and possible digestive health benefits. The review also discusses the challenges of aguamiel preservation and explores conventional and non-conventional technologies that can help conserve this valuable traditional beverage. By delving into the nutritional composition, health benefits, and conservation concerns surrounding aguamiel, we can fully appreciate its significance as a cultural and nutritional asset.

This review presents a comprehensive overview of aguamiel, including nutritional composition, microbial diversity, metabolic profile, and preservation techniques, fostering a greater understanding and appreciation of this unique Mexican beverage.

## 2. The Role of Aguamiel in National Production and Its Contribution to the Economy

Aguamiel has long been integral to Mexican culture and traditions [8,9]. However, its potential as an economic resource has often been overlooked [10]. In recent years, there has been a growing recognition of the significant role that aguamiel can play in both national production and the economy. Thus, for a long time, agave pulquero domestication has given rise to different variations that allow for improving the yield of aguamiel output, highlighting species such as *A. salmiana*, *A. americana*, *A. ferox*, *A. mapisaga*, *A. angustofilia*, *A. atrovirens*, among others [11]. In the Mexican territory in 2023, a planted area of 20,202.79 Ha was recorded, with a production of 399,535.25 tons and a production value of approximately 107,874.884 (thousands of USD) [12].

In Mexico, several states show exceptional agave production for aguamiel, with Oaxaca, Guanajuato, Michoacán, Jalisco, Guerrero, Puebla, Zacatecas, Colima, and the Estate of Mexico (Edomex) being the national leaders in production value (Table 1). These regions have ideal climatic conditions and a rich agricultural heritage, which favors the cultivation and harvesting of various agave species famous for their potential for aguamiel production. Recognized for their expertise in traditional techniques and commitment to quality, Mexican producers in these states continue to elevate the art of the aguamiel output, contributing significantly to the country’s rich culinary and cultural heritage [13].

## 3. Aguamiel and Its Composition

The nutritional composition of aguamiel is gaining the scientific community’s attention for its potential health benefits [14]. Among its characteristics, aguamiel, besides being a sweet and slightly viscous liquid extracted from the heart or center of the “piña” of different *Agave* species, is a very representative beverage of Mexican culture and gastronomy since pre-Hispanic times. Considered a sacred elixir by these populations, aguamiel is a refreshing drink and an essential source of nutrients, microorganisms, and bioactive compounds that potentially benefit human health [13,15]. However, before any other consideration, it is crucial to understand the physicochemical properties of the beverage. These properties are the basis for guaranteeing its quality and controlling variables such as pH, acidity, and sugar content that can satisfy consumer preferences.

### 3.1. Physicochemical Composition of Aguamiel

The genetic diversity of *Agave* plants allows them to be a natural source of a complex mixture of carbohydrates, vitamins, and minerals, which confer variations in the physicochemical properties of their sap (Table 2). These differences have significant implications for the quality and characteristics of derived products [16].

Previous studies have evaluated the composition of aguamiel in different species. *Agave* spp., with total soluble solids between 9.8 and 16 °Brix, lactic acid content between 0.03 and 1.03%, and pH between 4.3 and 7.5. On the other hand, species such as *A. salmiana* and *A. atrovirens*, commonly used for aguamiel production, show total soluble solids values between 9.3 and 14.11 °Brix.

The lactic acid content in these latter species is generally lower, with a range of 0.06 to 0.31%, and the pH is within a narrower range, between 5.53 and 6.29 [17,18,19]. Variations in the physicochemical parameters of aguamiel have been observed, and they are associated with the *Agave* genus’s unique ability to perform crassulacean acid metabolism (CAM). Unlike the typical photosynthesis in most plants, CAM allows agaves to capture carbon dioxide [20,21] efficiently. Other factors influencing aguamiel composition include photosynthetic radiation, geographic location, and seasonal variations. Winter and spring are generally periods of higher CO_2_ uptake for these plants. Furthermore, internal processes such as the hydrolysis of stored sugars and the synthesis of amino acids contribute to interspecific differences in aguamiel composition [2,16].

**Table 2:** Physicochemical parameters from aguamiel.

Species	Parameters			
	Total Soluble Solids (° Brix)	Acidity (% Latic Acid)	pH	Reference
*Agave* spp.	16	0.68 ± 1	4.3	[17]
*A. furcraea andina*	15 ± 0.10	--	6.00 ± 0.10	[22]
*A. americana*	--	0.03	7.72	[8]
*Agave* spp.	13–17	0.9–1.03	6.6–7.5	[3]
*Agave* spp.	9.8 ± 0.10	0.16 ± 0.01	5.3 ± 0.06	[18]
*A. atrovirens*	11.10 ± 0.10	0.06 ± 0.02	6.29 ± 0.02	[16]
*A. americana* L.	10.33 ± 0.03	0.319 ± 0.02	4.85 ± 0.05	[2]
*A. salmiana*	9.85 ± 1.08	--	5.73 ± 0.29	[23]
*A. atrovirens*	9.55 ± 0.15	--	6.00 ± 0.12	[23]
*A. salmiana*	14.11 ± 0.43	0.10 ± 0.006	5.53 ± 0.01	[24]
*Agave* spp.	11.70	--	6.43	[10]
*A. salmiana*	9.3 ± 0.5	--	6.2 ± 0.1	[19]
*A. salmiana*	9.8 ± 0.89	0.23 ± 0.04	6.23 ± 0.39	[25]
*A. salmiana*	13.33	0.21	4.37	[9]

### 3.2. Proximal Chemical Composition of Aguamiel

The proximal chemical composition of aguamiel varied among different species (Table 3). Moisture content remained relatively constant, ranging from 87% to 90.04% [8,26]. However, significant differences were observed in ash and protein levels, critical components for considering aguamiel as a nutritious beverage.

Results reported by Duque-Buitrago et al. [26] and Espíndola-Sotres et al. [24] indicate a range of 0.25 to 3.18 g/100 mL of protein for the genus *Agave* spp., with *A. Salmiana* showing the highest content.

A range of 0.13 to 0.54 g/100 mL was recorded regarding ash content. Additionally, the presence of an average of 6.44 g/100 mL of reducing sugars and 1.13 g/100 mL of fructooligosaccharides (FOS) indicates significant concentrations that suggest aguamiel could offer health benefits, thanks to the prebiotic properties of FOS [16]. FOS, classified as prebiotics, are selectively fermented by the colonic microbiota, leading to the generation of short-chain fatty acids. These microbial metabolites have been demonstrated to benefit host physiology, including lipid and glucose homeostasis modulation and enhancement of gastrointestinal mucosal barrier function [9,27].

**Table 3:** Proximal chemical composition from aguamiel extracted from *Agave* species.

Species	Parameters					
	Moisture (%)	Proteins (g/100 mL)	Ahs (g/100 mL)	Reducing Sugars (g/100 mL)	FOS (g/100 mL)	Reference
*Agave* spp.	--	0.49 ± 0.02	0.54 ± 0.03	2.26 ± 0.03	--	[17]
*A. atrovirens*	89.61 ± 0.02	0.36 ± 0.01	0.32 ± 0.02	6.37 ± 0.08	1.61 ± 0.01	[9]
*A. americana*	87.38	0.30	0.23	11.38 ± 0.3	--	[8]
*A. mapisaga*	88.5	0.35	--	6.78	1.17	[16]
*A. atrovirens*	--	0.39	--	6.81	1.72	[16]
*Agave* spp.	90.04 ± 0.11	0.25 ± 0.02	0.13 ± 0.03	8.09 ± 0.43	1.23 ± 0.06	[26]
*A. salmiana*	--	0.38 ± 0.07	--	2.17 ± 0.2	0.54	[23]
*A. atrovirens*	--	0.35 ± 0.04	--	3.56 ± 2.68	0.51	[23]
*A. salmiana*	--	3.18 ± 0.35	--	10.72 ± 0.16	--	[24]
*A. salmiana*	--	1.18	0.24	6.33	--	[28]

FOS: fructooligosaccharides.

On the other hand, knowing the vitamin content of aguamiel is vital to evaluating whether the beverage contributes to meeting some of the daily nutritional needs.

### 3.3. Vitamins of Aguamiel

**Figure 1:**
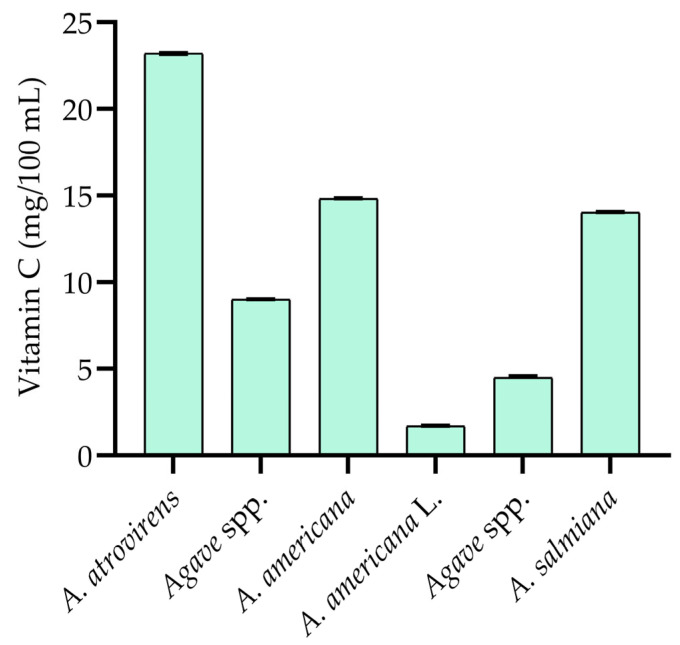
Content of vitamin C in aguamiel made from *Agave* species [2,3,8,9,26,30].

Aguamiel is a rich source of water-soluble vitamins, essential for optimal bodily functions [29]. These vitamins act as cofactors in various metabolic processes, from energy production to the synthesis of neurotransmitters. Due to their rapid excretion requires continuous dietary intake [3,8]. Previous studies [3,9] have identified thiamine, riboflavin, pyridoxine, and niacin in aguamiel (Table 4), with vitamin B3 showing the highest mean concentration of 6.15 mg/100 mL in *Agave* species. Vitamin C is also present in aguamiel (Figure 1), although its concentration (1.70–23.20 mg/100 mL) may vary depending on factors such as agave variety, growing conditions, and climate [30].

In addition, mineral content is part of a beverage’s nutritional profile and is relevant information to validate its possible functionality.

### 3.4. Minerals of Aguamiel

The mineral composition of aguamiel, a sap extracted from various *Agave* species, is highly variable, as reported by Bautista and Arias [8], Romero-López et al. [9], and Escalante et al. [2]. Potassium, magnesium, phosphorus, and calcium were found in high concentrations in the aguamiel of *A. americana*, *A. atrovirens*, and other *Agave* species (Table 5). These variations can be attributed to factors such as cultivation practices, plant physiological status, and the specific agave species, given that over 200 species have been documented [16].

Other vital components in aguamiel are non-essential and essential amino acids.

### 3.5. Aminoacids of Aguamiel

Aguamiel is recognized as a nutritious beverage, mainly due to its amino acid content. Studies by Romero-López et al. [9] and Hernández-González et al. [31] have identified a wide range of essential and non-essential amino acids in this beverage, including histidine, arginine, phenylalanine, lysine, tyrosine, and valine (Table 6). These compounds are the building blocks of proteins and play vital roles in the body, from tissue repair to neurotransmitter synthesis. By providing a natural source of essential amino acids, aguamiel can contribute to a balanced diet and optimal health [9].

On the other hand, amino acids are identified as valuable compounds due to their relationship with antioxidant activity, a property associated with phenolic compounds and also found in aguamiel drinks.

### 3.6. Total Soluble Phenolics and Antioxidant Capacity of Aguamiel

According to various studies, Table 7 reveals a considerable range of total soluble phenols content (38.23 to 90.48 mg gallic acid equivalents/100 mL) and antioxidant capacity in aguamiel. Despite this variability, the results converge on a critical point: aguamiel emerges as a potentially rich source of compounds with antioxidant properties. These compounds are fundamental in neutralizing free radicals and mitigating oxidative stress associated with various chronic diseases, including cancer, cardiovascular diseases, and neurodegenerative diseases [9,26,30,32].

In addition to the nutrients mentioned above, aguamiel is characterized by the great diversity of microorganisms that can develop effectively in this food matrix.

## 4. Microbial Consortia in Aguamiel

Microbial consortia in aguamiel are crucial in converting maguey sugars into a wide range of volatile and non-volatile compounds that give aguamiel its characteristic flavor and aroma. In addition, microbial activity in aguamiel can influence its texture and shelf life. Some microorganisms can produce enzymes that break down complex carbohydrates in aguamiel, resulting in a thinner and more fluid consistency [23,28].

A microbiological analysis of aguamiel derived from *A. atrovirens* and *Agave salmiana* revealed the presence of diverse microbial communities. The microorganisms identified included *Acetobacter* sp., *Acetobacter pasteurianus*, *Lactobacillus* spp. (*L. plantarum* and *L. casei*) and *Leuconostoc* spp., as well as yeasts such as *Clavispora* sp., *Candida* sp., *Saccharomyces* sp., and *Kluyveromyces* sp. [5,23,33].

A wide range of bacteria has been documented in aguamiel obtained from *Agave* spp. These bacteria are consistently present regardless of the collection period and include microorganisms such as *Lactococcus* spp., *Pediococcus* spp., *Trichococcus* spp., *Kazachstania zonata* and *Kluyveromyces marxianus*. In addition, the presence of specific strains such as *L. acidophilus*, *L. kefir*, *L. acetotolerans*, *L. hilgardii*, *L. plantarum*, *Leuconostoc mesenteroides*, *L. pseudomesenteroides*, *Microbacterium arborescens*, *Flavobacterium johnsoniae*, *Acetobacter pomorum*, *Gluconobacter oxydans*, and *Hafnia alvei* has been reported [34].

Other studies have revealed the presence of lactic acid bacteria such as *Lactobacillus* sp., yeasts such as *Kluyveromyces* sp. and *Saccharomyces cerevisiae*, exopolysaccharide-producing *Leuconostoc* sp. and ethanol-producing *Zymomonas mobilis* [33]. Another analysis of *A. salmiana* revealed the presence of *Lactobacillus* and *Leuconostoc* spp. In addition, a wide variety of yeasts, including *Saccharomyces cerevisiae*, *Kluyveromyces,* and *Zymomonas mobilis*, as well as acetic acid bacteria such as *Acetobacter* and *Gluconobacter*, were detected in the analyzed sample [28]. Escalante et al. [35] reported that the predominant bacteria identified in the aguamiel were species of *Lactobacillus* spp., specifically *L. mesenteroides*, *L. citreum*, related to *L. acidophilus*, the proteobacterium *Acinetobacter radioresistens*, *Erwinia rhapontici*, *Enterobacter* spp., *Kluyvera ascorbata*, *Serratia grimensis*, and the acetic acid bacteria *Acetobacter malorum* [35].

Another study indicated that microorganisms such as *L. acetotolerans*, *L. brevis*, *L. camelliae,* and *L. hilgardii* were found in the fresh aguamiel. As the fermentation stages progressed, *Lactobacillus senioris* and *L. similis* were detected. Regarding fungi, the presence of *Aureobasidium pullulans*, *Penicillium* sp., *Torulaspora delbrueckii*, *Rectipilus* sp., *Malassezia globosa*, *M. restricta*, *Botrytis caroliniana*, *Erythrobasidium hasegawianum,* and *Naganishia albida* was identified [36].

As mentioned above, aguamiel is rich in nutrients and microorganisms, making it susceptible to rapid fermentation, which modifies metabolites and nutrients.

## 5. Metabolites and Nutrients Derived from Aguamiel Fermentation

Fermentation produces various metabolites, such as ethanol, organic acids, and aromatic compounds. These components contribute to the unique flavor profile of aguamiel and may offer potential health benefits, such as improved gut health and enhanced antioxidant protection [5,28,33].

Ethanol is the leading alcoholic product derived from the fermentation of aguamiel. Various microorganisms, including *Saccharomyces cerevisiae*, *Zymomonas*, and *Lactobacillus* spp., catalyze this anaerobic process. The monosaccharides fructose and glucose in aguamiel serve as substrates for these microorganisms, which metabolize them to produce ethanol and carbon dioxide as primary products [37].

Lactic (*Lactobacillus* spp.) and acetic (*Acetobacter* spp.) bacteria bioconvert aguamiel sugars into organic acids, such as lactic and acetic [3]. These acids contribute to the preservation of aguamiel and significantly influence its sensory attributes. The microbial community involved in this process generates a diverse range of organic acids, such as succinic and citric, which collectively contribute to the complexity and balance of the flavor profile of the final product [25].

Fermentation of aguamiel is a biochemical process that, in addition to producing alcohol, enriches the final product with essential amino acids, which are vital for human health and contribute to the nutritional value of pulque. Proteases produced by yeasts and bacteria catalyze the hydrolysis of aguamiel proteins, releasing peptides and free amino acids [28]. In turn, microorganisms use these as a source of nitrogen for their growth and metabolism. Some yeasts, such as *Saccharomyces cerevisiae*, possess biosynthetic pathways for producing amino acids, further contributing to the enrichment of fermented beverages [9,31].

Although most of the vitamins come from the *Agave* plant, fermentation of aguamiel can also result in the synthesis of vitamins, particularly those belonging to the B complex and vitamin K [15]. The microorganisms involved in this process, such as lactic acid bacteria and yeasts, possess the enzymatic activity necessary for vitamin biosynthesis. Synergistic interactions between microbial species can enhance these biosynthetic pathways, further enriching the final product [3,9].

On the other hand, fermentation of aguamiel results in increased production of short-chain fatty acids (SCFA) through the metabolic activities of microorganisms. Lactic acid bacteria and some yeasts use various metabolic pathways to convert the carbohydrates in aguamiel into SCFA, such as acetic, propionic, and butyric acids. These SCFAs play a crucial role in shaping the sensory attributes of aguamiel, contributing to its acidity and flavor profile. In addition, SCFA has been associated with numerous health benefits, such as promoting intestinal health and exerting anti-inflammatory effects [37,38]. Anaerobic fermentation of aguamiel is characterized by activating specific metabolic pathways, such as glycolysis and the pentose phosphate pathway, which facilitate the production of short-chain fatty acids (SCFA). The resulting SCFA has been shown to benefit intestinal health, serving as a primary energy source for colonocytes and modulating inflammatory responses [26,27].

In addition, the fermentation process of aguamiel allows the breakdown of complex carbohydrates into simpler sugars, increasing polysaccharide content. This is because yeasts and bacteria produce polysaccharides during fermentation as part of their metabolism [39]. For example, *Zymomonas mobilis*, a common bacterium in aguamiel fermentation, is known for its ability to synthesize extracellular polysaccharides, such as dextrans and fructans, which can increase the viscosity and polysaccharide content of the final product. In addition, some sugars can be converted into polysaccharides. For example, fructose fermentation can result in the formation of fructans through the action of specific enzymes (sucrose: sucrose-1-fructosyltransferase, fructan: fructan 1-fructosyltransferase, fructan: fructan 6-G-fructosyltransferase) [25,40].

The presence of live microorganisms, mainly lactic acid bacteria and yeasts, in the fermented aguamiel product can provide probiotic benefits, promote intestinal health, and improve the immune system. These metabolites and nutrients enhance flavor and health benefits and reflect the complex microbial interactions during aguamiel fermentation [33,41,42]. During fermentation, environmental conditions (such as temperature, pH, and nutrient availability) favor the growth of beneficial microorganisms, such as certain species of lactic acid bacteria and yeasts. Probiotics in aguamiel can positively affect digestive health, improving intestinal flora and modulating the immune system [6,34,43].

On the other hand, the presence of exopolysaccharides (EPS), produced by microorganisms such as *Leuconostoc mesenteroides*, *Leuconostoc dextranicum*, and genera such as *Lactobacillus* and *Lactococcus* during fermentation, can influence the viscosity and texture of aguamiel. Their synthesis increases due to various factors related to the activity of the microorganisms and the conditions of the fermentation process. During fermentation, certain bacteria, especially lactic acid bacteria, and some *Leuconostoc* species can synthesize exopolysaccharides as part of their metabolism [44,45]. These microorganisms use the sugars available in the medium to produce EPS. Exopolysaccharides have several functions in microbial metabolism, such as protection against adverse conditions, formation of biofilms, and improvement of the viscosity of the medium [46]. In addition, a lower pH or the presence of certain nutrients can favor the synthesis of exopolysaccharides by bacteria, possessing functional properties that can improve the quality of the final product. For example, they can act as thickening agents, improve the texture and stability of fermented aguamiel, and have prebiotic effects that benefit the intestinal health of the consumer [47,48].

Furthermore, during the fermentation process, an increase in saponin biosynthesis has been demonstrated, as microorganisms can activate metabolic pathways (mevalonic acid pathway, methylerythritol phosphate (MEP) pathway, shikimate pathway, sugar biosynthesis pathways) that favor the production of saponins [49]. These molecules are bioactive compounds derived from steroids or triterpenoids. Fermentation can modify the availability of precursor compounds (isoprenoids, glucose, galactose, arabinose, xylose, and amino acids) necessary for synthesizing saponins. Saponins possess biological properties that may benefit health, such as anti-inflammatory, hypolipidemic, and antioxidant effects [3].

In addition, FOS are prebiotic compounds that can contribute to intestinal health and improve the microbial community of the consumer. The increase of this compound during aguamiel fermentation has been related to specific microorganisms, such as some species of lactic acid bacteria and yeasts, which can produce fructooligosaccharides as part of their metabolism [50]. These microorganisms can use simple sugars, such as fructose and glucose, to synthesize FOS, short chains of fructose linked by glycosidic bonds. Some microorganisms present in aguamiel (*Kluyveromyces marxianus*, *Hanseniaspora uvarum*, *Saccharomyces cerevisiae*, *Aspergillus niger*) can produce enzymes such as inulinase, which acts on inulin to release FOS [51,52]. These enzymes can be induced during fermentation, resulting in increased production of fructooligosaccharides. Fructooligosaccharides are considered prebiotics, which can stimulate beneficial microorganisms’ growth and activity in the gut. Fructooligosaccharides identified in aguamiel include 1-costrose (GF2), nystose (GF3), and 1F-β-fructofuranosylnystose (GF4). These compounds are part of the fructose-derived fructooligosaccharide series and are known for their prebiotic effects [53,54]. Other benefits have also been related to human health.

## 6. Effects of Aguamiel on Human Health

Recent research has highlighted the probiotic and prebiotic properties of aguamiel, suggesting that it may contribute to intestinal health, stimulate immune activity, and provide antioxidant protection [3,28,30,55].

The composition of aguamiel and its metabolites reveals a complex matrix of nutrients and bioactive compounds with potential health benefits (Table 8) [33,43]. For example, it has been shown that microorganisms, such as *Leuconostoc* and *Lactobacillus*, resist the antimicrobial barriers of the gastrointestinal tract and adhere to the intestinal mucosa; this ability may contribute to antimicrobial activity and potentially decrease infections and gastrointestinal disorders after consumption of aguamiel and pulque [3,28,43,56]. Phenolic compounds and saponins present in aguamiel exhibit anti-inflammatory effects by inhibiting the production of proinflammatory mediators such as vasoactive amines, eicosanoids, and cytokines. This reduction of inflammatory mediators contributes to a decreased inflammatory response to various insults, including infections and injuries [37]. Aguamiel has been shown to have a promising ability to selectively reduce serum total cholesterol levels without adversely affecting glucose metabolism. In a group of hypercholesterolemic adult men with baseline total cholesterol levels ranging from 263 mg/dL, aguamiel consumption decreased these levels to 141.6 mg/dL. These results indicate that aguamiel may be a possible dietary intervention for hypercholesterolemia [57].

Another study showed that consuming aguamiel can treat kidney failure, coughs, and colds and control anemia. It also helps to improve the absorption of essential minerals such as calcium and iron. In addition, it has been shown to lower cholesterol, exhibit antioxidant properties, and may have protective effects against colon cancer [16,58,59].

The strains of lactic acid bacteria isolated from aguamiel (*Lactobacillus plantarum* and other species of the genus *Lactobacillus* and *Pediococcus*) exhibited significant antibacterial properties, showing activity against pathogens such as *E. coli*, *S. aureus,* and *H. pylori*. All strains evaluated inhibited the urease activity of *H. pylori*, which may decrease its survival in the stomach, suggesting their potential as natural antimicrobial agents [6,60].

**Table 8:** Studies on potential benefit of aguamiel consumption.

Biological Activity	Model	Concentration or Doses	Results	Reference
Attenuated obesity and hepatic steatosis	Male C57BL/6 mice 5 weeks old 17–22 g	Aguamiel concentrate (2.8 g/kg of diet), over 12 weeks	The mice showed a significant decrease in weight gain compared to the high-fat diet group, and reductions in serum glucose, insulin, and LDL cholesterol levels were observed.	[14]
Hypercholesterolemic	Group I: two young men (22–23 years old) Group II: three women (48–60 years old) Group III: four men (48–55 years old)	250 mL of aguamiel every three days, over 35 days	After aguamiel intake in hyperlipidemic men, cholesterol decreased from 263 mg/dL to optimal levels (less than 200 mg/dL).	[57]
Cancer antiproliferative	Colorectal cancer cell line Caco-2 (HTB-37, ATCC)	Fraction rich in protodioscine extracted from aguamiel (25 μg/mL)	The protodiscine fraction showed a significant antiproliferative effect, reducing cell viability to 50%.	[58]
Hematic biometry	Nine Landrance male rabbits 55 days old 965–1153.3 g	250 mL boiled aguamiel, or 250 mL fresh aguamiel daily, over 60 days	The best treatment was boiled aguamiel, which promoted an 8% increase in rabbit weight compared to the control group. In addition, a 9% increase in hemoglobin content and a 5.3% increase in hematocrit were observed in the rabbits that received boiled aguamiel.	[30]
Antimicrobial	*E. coli* ATCC 25922, *P. aeruginosa* ATCC 27853, *S. aureus* ATCC 25923	*Lactobacillus paracasei* KSI extracted from aguamiel	The extract exhibited a MIC of 350 μg/mL against *E. coli* and *S. aureus*, and 700 μg/mL against *P. aeruginosa*.	[60]
Antimicrobial	*Staphylococcus aureus* ATCC 29213, *Escherichia coli* ATCC 25922	*Lactobacillus plantarum* and *Pediococcus acidilactici* isolated from aguamiel	Lactic acid bacteria isolated from aguamiel showed antimicrobial activity as inhbition zone) for *E. coli* (24–30 mm) and *S. aureus* (21–22 mm).	[6]
Anticancer	Hep-G2 (liver cancer) and Caco-2 (colon cancer) cell lines	50 μg/mL of saponin-rich extracts from aguamiel	A reduction in Hep-G2 viability from 81.4% to 66.6% and in Caco-2 viability from 70.2% to 56.0% was observed.	[59]
Gastroprotective	36 female Wistar rats 4.5 weeks old 180 g	Aguamiel adjusted to two doses of fructooligosaccharides (100 and 200 mg FOS/kg)	The reduction of gastric lesions with a dose of 200 mg FOS/kg (1647.84 µm^2^) was comparable to that of ranitidine (1546.16 µm^2^).	[56]

MIC = Minimum Inhibitory Concentration.

In addition, aguamiel has a low glycemic index, suggesting its potential as a hypoglycemic agent. Several bioactive compounds, including phenolic compounds and saponins, contribute to its α-glucosidase inhibitory activity. This mechanism of action may benefit people with diabetes by modulating postprandial glucose variations and improving insulin sensitivity [26]. Likewise, aguamiel is rich in highly digestible carbohydrates and essential minerals such as iron and zinc. Consuming 850 mL of aguamiel can satisfy humans’ daily requirement for iron and zinc. In addition, it contains essential amino acids and γ-aminobutyric acid (GABA), which may contribute to its health benefits [3,13].

Ortiz-Basurto et al. (2008) suggested that aguamiel is a functional beverage due to its GABA content, which may help reduce blood pressure in people with mild hypertension.

An independent study on *Agave salmiana* extract rich in saponins demonstrated beneficial effects in obese mice, further reducing key metabolic markers such as glucose, insulin, and LDL cholesterol. These cumulative findings reinforce the potential of saponins as bioactive compounds for obesity management [14].

Nonetheless, consistent consumption of aguamiel could be limited for individuals with diabetes, hypertension or both who are on medication. This is due to the low glucose content or mineral composition (potassium and magnesium) from aguamiel. The combination of these medications with aguamiel consumption could lead to hypoglycemia or hypotension [61,62]. However, to date, there is no scientific evidence to indicate the dose of aguamiel/day that could cause these issues in individuals with these conditions or whether occasional consumption of this beverage poses no risk. Therefore, this represents an excellent perspective for future research.

Despite all the benefits found in aguamiel, it is crucial to keep in mind that the shelf life of aguamiel is highly reduced, and this complicates its availability to the population, so it is necessary to explore technologies that help to have a commercially accessible beverage for an extended period of time. The following are the technologies studied so far to preserve aguamiel.

## 7. Preservation Technologies of Aguamiel

In the past, very little research has focused on applying conventional aguamiel preservation technologies to prolong the shelf life of aguamiel. However, pasteurization has been reported to be one of the technologies employed in food processing [63]. According to a study by Chagua-Rodriguez et al. [2], the optimal pasteurization time should be less than 10 min at a temperature of 80 °C. Although this process increases reducing sugars in the range of 11.38% to 25.39%, it significantly reduces β-carotene and vitamin C concentrations. Table 9 shows the characteristics of the technologies used to extend the shelf life of aguamiel and its derivative (pulque). For example, conventional pasteurization at 63 °C for 30 min has been applied to preserve pulque; however, this technique does not maintain all the quality parameters of pulque, such as color and sensory properties [5].

In recent years, more needs to be explored regarding using new technologies to improve aguamiel’s quality and shelf life. One of them is microfiltration; this was applied using a Pellicon 2 Mini cross-flow filtration module (Millipore, St. Louis, MO, USA) using a Pellicon 2 Mini, Biomax-10 (Millipore, St. Louis, MO, USA) microfiltration membrane cartridge with a nominal pore size of 0.45 μm. However, reducing the load to 2.89 log CFU/mL was possible without negatively affecting its physicochemical attributes. This means that the essential qualities of aguamiel, such as flavor and nutritional content, are maintained. Nonetheless, in this work, the shelf life of the treated aguamiel was not evaluated, so it is difficult to predict the effectiveness of the treatment [18]. On the other hand, the results indicate that thermosonication, applied at amplitudes of 75% for 6–9 min and 85% for 4–6 min, is a promising technique for preserving pulque. These conditions allow for maintaining the viability of lactic acid bacteria and yeasts, which are responsible for the organoleptic and functional characteristics of the product, but only extend its shelf life up to 24 days at 4 °C [5].

Applying ohmic heating (80 °C, 200 V for 5 s) to the aguamiel resulted in a significant logarithmic reduction of *E. coli*, yeasts, and lactobacilli, leading to complete microbial inactivation. Physicochemical analysis showed that this technology does not negatively affect product quality, although a decrease in pH and color of the beverage was observed, and brightness was affected. These findings indicate that ohmic heating could be an alternative for the preservation of aguamiel; however, the shelf life of the treated aguamiel was not evaluated, which limits its effectiveness [19].

As for the application of high pressures (400 MPa, 4 min) to the aguamiel, this technology allowed a remarkable reduction of the microbial load, as evidenced by the significant logarithmic reduction of aerobic mesophiles, coliforms, yeasts, and molds. While these findings suggest potential for improving product shelf life, the microbial levels achieved did not meet the quality standards established by the FDA [64].

On the other hand, it was reported that tangential microfiltration with a ceramic membrane system and pore size of 0.2 µm (Pall Membralox, Port Washington, WI, USA), operating at a pressure of 2 kg/cm^2^, is a method that allows reducing the microbial load in the aguamiel, achieving approximately 1.40 and 1.18 log UCF/mL reduction in aerobic mesophilic bacteria and in molds and yeasts, respectively. However, this process may eliminate beneficial microorganisms in aguamiel, such as *Lactobacillus*, suggesting the possible loss of probiotic properties [65]. In this study, the shelf life of treated aguamiel was not determined, so further study would be necessary to confirm the prolonged efficacy of microfiltration as a preservation method.

López-Martínez (2018) inferred that thermosonication is an efficient method for microbial inactivation, combining the effects of heat and cavitation. This procedure allows a significant microbial reduction at lower temperatures and shorter treatment times, preserving the sensory quality of the aguamiel. The optimum parameters of the process were determined as a temperature of 51 ± 1 °C, a treatment time of 19 min, and an ultrasound amplitude of 80%; however, the project was limited to evaluating the stability of the beverage under the conditions mentioned above (reaching 88.14% stability), so it is not possible to estimate the appropriate storage time [66].

## 8. Conclusions

Aguamiel, a traditional Mexican beverage derived from the sap of maguey plants, offers a unique combination of nutritional and sensory properties. It is a rich source of essential nutrients, such as vitamins, minerals, amino acids, and fructooligosaccharides, with prebiotic potential. The fermentation process of aguamiel increases its nutritional value by producing metabolites such as organic acids, short-chain fatty acids, and polysaccharides. In addition, fresh aguamiel harbors beneficial microorganisms, such as lactic acid bacteria and yeasts, which contribute to its probiotic properties and make it a promising source of bioactive compounds with potential applications in the food and nutraceutical industries. However, the susceptibility of aguamiel to rapid fermentation poses problems for its preservation. Several methods have been investigated to prolong its shelf life while preserving its nutrients and prebiotic properties. Further research is needed to explore new preservation techniques and to fully understand the long-term effects of fermentation on the nutritional and functional properties of aguamiel. Furthermore, by promoting the sustainable production and consumption of aguamiel, we can contribute to the conservation of Mexican cultural heritage and the development of innovative functional foods.

## Figures and Tables

**Table 1:** Agricultural production of *Agave plants* in Mexico [12].

States	Planted Area (Ha)	Harvested Area (Ha)	Production (Ton)	Harvested Obtained (Ton/Ha)	Production Value (Thousands of USD)
Oaxaca	11,736.09	3683.38	256,549.04	69.67	48,357.732
Guanajuato	4573.00	948.00	56,476.00	59.57	46,551.624
Michoacán	2101.00	756.00	58,197.89	76.98	29,504.931
Jalisco	770.00	186.00	13,739.00	73.87	10,557.938
Guerrero	1668.80	957.30	36,134.87	37.75	9440.571
Puebla	2923.00	221.00	20,246.04	91.61	3594.460
Zacatecas	112.00	22.00	1980.04	90.00	1401.895
Colima	86.29	76.29	5025.24	65.87	1,275,214
Edomex	63.00	63.00	4554.90	72.30	1165.764

**Table 4:** Content of B vitamins from aguamiel extracted from *Agave* species.

Species	B Vitamins (mg/100 mL)				
	Thiamine (B1)	Riboflavin (B2)	Niacin (B3)	Pyridoxine (B6)	Reference
*A. atrovirens*	0.12 ± 0.03	0.49 ± 0.11	6.15 ± 0.17	0.73 ± 0.08	[9]
*Agave* spp.	0.60	20	450	22.98	[3]

**Table 5:** Minerals from aguamiel extracted from different *Agave* species.

Minerals (mg/100 mL)	Species		
	*A. atrovirens*	*A. americana*	*Agave* spp.
Potassium (K)	155.38 ± 0.01	14.56	--
Calcium (Ca)	15.09 ± 0.03	9.72	20
Lead (Pb)	0.018 ± 0.00	--	--
Zinc (Zn)	0.23 ± 0.01	0.07	1.41
Iron (Fe)	1.02 ± 0.02	0.06	2.15
Sodium (Na)	0.10 ± 0.06	5.92	--
Copper (Cu)	0.90 ± 0.03	0.02	0.74
Magnesium (Mg)	0.69 ± 0.07	8.60	10
Selenium (Se)	0.059 ± 0.00	--	--
Phosphorus (P)	--	4.20	20
Reference	[9]	[8]	[3]

**Table 6:** Amino acid profile of aguamiel extracted from different *Agave* species.

Amino Acids (mg/100 mL)	Species	
	*A. atrovirens*	*A. atrovirens* Karw
Aspartic acid	0.99 ± 0.06	--
Glutamic acid	2.53 ± 0.02	--
Serina	0.56 ± 0.01	--
Glycine	0.31 ± 0.08	--
Histidine	0.23 ± 0.04	0.04
Arginine	3.97 ± 0.09	0.04
Threonine	0.50 ± 0.06	--
Alanine	0.30 ± 0.04	--
Proline	0.93 ± 0.03	--
Tyrosine	0.45 ± 0.01	0.23
Valine	2.06 ± 0.04	0.13
Methionine	0.27 ± 0.06	0.08
Cysteine	0.06 ± 0.01	--
Isoleucine	0.59 ± 0.02	--
Leucine	0.58 ± 0.03	0.09
Phenylalanine	1.26 ± 0.09	0.25
Lysine	0.65 ± 0.04	0.13
Reference	[9]	[31]

**Table 7:** Total soluble phenolics and antioxidant capacity of aguamiel.

Species	Total Phenols	Antioxidant Capacity			Reference
		ABTS	DPPH	FRAP	
*A. atrovirens*	38.23 ± 5.93 *	112.06 ± 2.52 **	110.04 ± 1.26 **	--	[9]
*Agave* spp.	--	87.3 ± 7.02 ^x^	--	--	[26]
*A. salmiana*	--	--	34.81 ± 5.75 **	42.65 ± 5.85 **	[32]
*A. salmiana*	90.48 *	--	109.8 **	--	[30]

* mg gallic acid equivalent/100 mL; ** mmol Trolox equivalent/100 mL; ^x^ mol Trolox equivalent/100 mL.

**Table 9:** Use of different technologies for preservation of aguamiel.

Sample	Technology	Conditions	Reduced Microorganisms	Initial Load (Log CFU/mL)	Log Reduction (Log CFU/mL)	Reference
Pulque	Pasteurization	10 min 63 °C	Lactic acid bacteria	7.77	4.13	[5]
Aguamiel	Microfiltration	0.45 μm pore size of membrane	Aerobic mesophiles	5.7	2.89	[18]
Pulque	Thermosonication	95% 9 min	Lactic acid bacteria	7.77	5.85	[5]
Aguamiel	Ohmic heating	80 °C, 200 V for 5 s	*E. coli* Yeasts Lactobacilli	5.48 5.46 7.73	Inactivation	[19]
Aguamiel	High hydrostatic pressure	400 MPa 4 min	Aerobic mesophiles	6.76	2.90	[64]
Aguamiel	Microfiltration	0.2 µm pore size	Aerobic mesophiles	2.40	1.40	[65]
Aguamiel	Thermosonication	51 ± 1 °C amplitude 80%	Aerobic mesophiles	4.97	0.78	[66]

## Data Availability

No new data were created or analyzed in this study. Data sharing is not applicable to this article.
